# A Novel Spectrophotometric Method for Determination of Percarbonate by Using N, N-Diethyl-P-Phenylenediamine as an Indicator and Its Application in Activated Percarbonate Degradation of Ibuprofen

**DOI:** 10.3390/molecules28237732

**Published:** 2023-11-23

**Authors:** Jinying Li, Aoxue Chen, Qingling Meng, Honghai Xue, Baoling Yuan

**Affiliations:** Key Laboratory of Songliao Aquatic Environment, Ministry of Education, Jilin Jianzhu University, Changchun 130118, China; 15840249352@163.com (J.L.); chenaoxue2002@163.com (A.C.); mengql1984@163.com (Q.M.); yuanbl@hotmail.com (B.Y.)

**Keywords:** advanced oxidation process, determination, N, N-diethyl-p-phenylenediamine, percarbonate, ultraviolet–visible spectrophotometry

## Abstract

Sodium percarbonate (SPC) concentration can be determined spectrophotometrically by using N, N-diethyl-p-phenylenediamine (DPD) as an indicator for the first time. The ultraviolet–visible spectrophotometry absorbance of DPD^•+^ measured at 551 nm was used to indicate SPC concentration. The method had good linearity (R^2^ = 0.9995) under the optimized experimental conditions (pH value = 3.50, DPD = 4 mM, Fe^2+^ = 0.5 mM, and t = 4 min) when the concentration of SPC was in the range of 0–50 μM. The blank spiked recovery of SPC was 95–105%. The detection limit and quantitative limit were 0.7–1.0 μM and 2.5–3.3 μM, respectively. The absorbance values of DPD^•+^ remained stable within 4–20 min. The method was tolerant to natural water matrix and low concentration of hydroxylamine (<0.8 mM). The reaction stoichiometric efficiency of SPC-based advanced oxidation processes in the degradation of ibuprofen was assessed by the utilization rate of SPC. The DPD and the wastewater from the reaction were non-toxic to *Escherichia coli*. Therefore, the novel Fe^2+^/SPC-DPD spectrophotometry proposed in this work can be used for accurate and safe measurement of SPC in water.

## 1. Introduction

Sodium percarbonate (SPC) is a substitute for hydrogen peroxide (H_2_O_2_). SPC has several advantages over liquid H_2_O_2_ including low price, wide pH application, good thermal stability, water solubility, and easier storage and transportation [1,2]. It has typically been used as a bleach and fungicide in the textile and paper industries [3], and recently it was applied in the SPC-based advanced oxidation process (AOPs) to improve water environments [4]. For example, Fe^2+^ activated SPC can produce hydroxyl radical (·OH) (E^0^(∙OH/OH^−^) = 2.74 V) [5] and ·OH can oxidize organic pollutants from water (Equations (1) and (2)) [6,7]. Therefore, it is needed to accurately determine the consumption of SPC during the degradation of contaminants in SPC-based AOPs. This can obtain the optimal SPC dosage, and avoid introducing excessive SPC into the water environment.

(1) $${\mathrm{Na}}_{2}{\mathrm{CO}}_{3}\cdot 1{.5H}_{2}O_{2}\;\to {\;2\mathrm{Na}}^{+}{+\mathrm{CO}}_{3}^{2-}+1{.5H}_{2}O_{2}$$

(2) $$H_{2}O_{2}{+\mathrm{Fe}}^{2+}\;\to {\;\mathrm{Fe}}^{3+}+∙{\mathrm{OH}+\mathrm{OH}}^{-}$$

Until now, several studies reported the determination methods of SPC. For example, Wada et al. [8] qualitatively analyzed the chemical composition of SPC via iodometric titration, which is the standard determination method of SPC. However, the titration procedure is time-consuming and inconvenient for routine applications. Kang et al. [9] used the fluorometric method to detect SPC; the limit of detection was 0.45 μM and the recovery of SPC was 96.2–99.6%. Although the fluorometric method had high sensitivity, the experimental equipment was expensive and complex to operate. Recently, spectrophotometry has been considered a promising and advantageous method in recent years due to its rapid detection, low cost, and easy operation. Zuo et al. [10] established an iodometric spectrophotometry method to determine SPC in a microwave–ultraviolet/SPC system based on the characteristic peak of yellow I^3−^ at 352 nm generated from KI. However, the iodometric spectrophotometry method was susceptible to the components of natural water, and hazardous wastewater containing KI was produced [11]. Therefore, it is necessary to find an alternative indicator to realize an environmentally friendly spectrophotometric determination of SPC since SPC-based AOPs have been used in water treatment in recent years.

N, N-Diethyl-p-phenylenediamine (DPD) is an environmentally friendly indicator that was previously used for the determination of H_2_O_2_, hypochlorite, persulfate, and permanganate in water under peroxidase activation, Fe^2+^ activation, or thermal activation conditions [12,13,14,15,16]. This was because different catalytic systems can all produce reactive oxygen species (e.g., ·OH) with the ability to oxidize DPD to DPD^•+^ [13,17]. DPD^•+^ can be measured via ultraviolet–visible (UV-Vis) spectrophotometry since it has a strong absorption at wavelengths of 510 nm and 551 nm. As a strong oxidant, SPC has similar chemical properties to H_2_O_2_. In the presence of Fe(II), H_2_O_2_ produced by SPC can also oxidize the colorless DPD to pink DPD^•+^ via single electron transfer (Equation (3)). To the best of our knowledge, this is the first report on the determination of SPC using the proposed DPD spectrophotometry method.

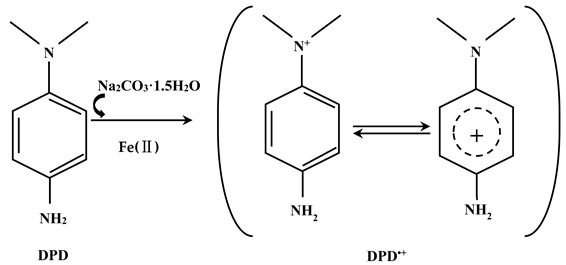
(3)

In this work, a novel method for the determination of SPC in water was designed and developed by using non-toxic DPD as an indicator and Fe^2+^ as a catalyst; hereinafter, we refer to the method as Fe^2+^/SPC-DPD spectrophotometry for short. The aims of this study were as follows: (1) to establish the linearity range of the method and to assess the stability of DPD^•+^; (2) to optimize experimental parameters (i.e., pH value, DPD, and Fe^2+^ concentration); (3) to observe the effects of natural water matrix and the existence of hydroxylamine (HA); (4) to obtain the utilization rate of SPC in two SPC-based AOPs in the degradation of ibuprofen; and (5) to assess the toxic effect of DPD and the wastewater from this method on *Escherichia coli* (*E. coli*) cell activity.

## 2. Results

### 2.1. UV-Visible Absorption Spectra of DPD^•+^

**Figure 1:**
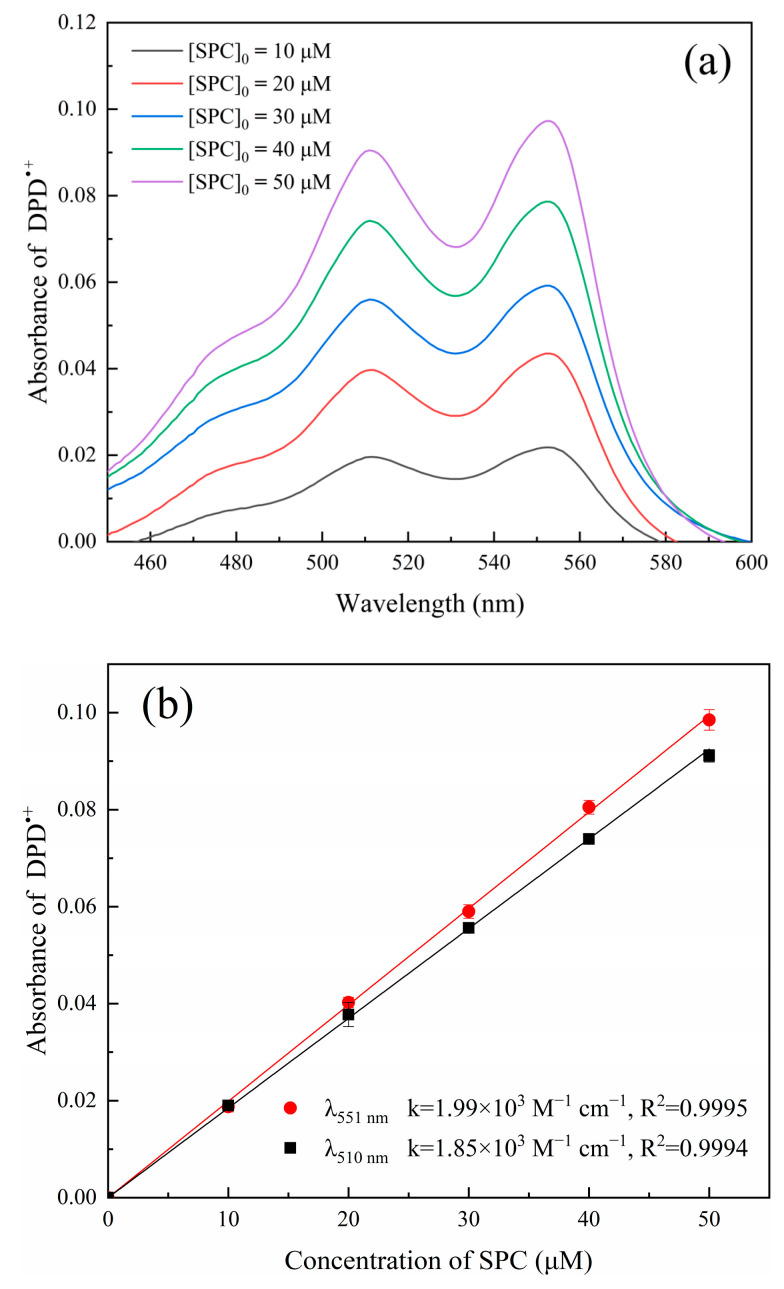
(**a**) Absorption spectra of DPD^•+^ with varied concentrations of SPC; (**b**) calibration curves for the determination of SPC concentration at wavelengths of 510 nm and 551 nm. ([DPD]_0_ = 4 mM, [Fe^2+^]_0_ = 0.5 mM, pH value = 3.50, t = 4 min, and T = 25 °C).

DPD can react with SPC with the existence of Fe^2+^, producing DPD^•+^ whose absorption spectra are shown in Figure 1a. Both 510 nm and 551 nm are the characteristic absorption peaks of DPD^•+^. As shown in Figure 1b, the absorbance of DPD^•+^ showed good linearity at 510 nm and 551 nm (R^2^ = 0.9995) with SPC in the concentration range of 0–50 μM; therefore, both 510 nm and 551 nm can be used to determine SPC. Finally, we chose 551 nm as the optimal detection wavelength because it has higher sensitivity (1.99 × 10^3^ M^−1^ cm^−1^) than 510 nm (1.85 × 10^3^ M^−1^ cm^−1^).

### 2.2. Optimization of SPC Determination Conditions

The solution pH value, initial concentration of DPD, and Fe^2+^ were key factors affecting the oxidation reaction time and stability of DPD^•+^. Therefore, the effects of these factors were studied in detail.

#### 2.2.1. pH Value

**Figure 2:**
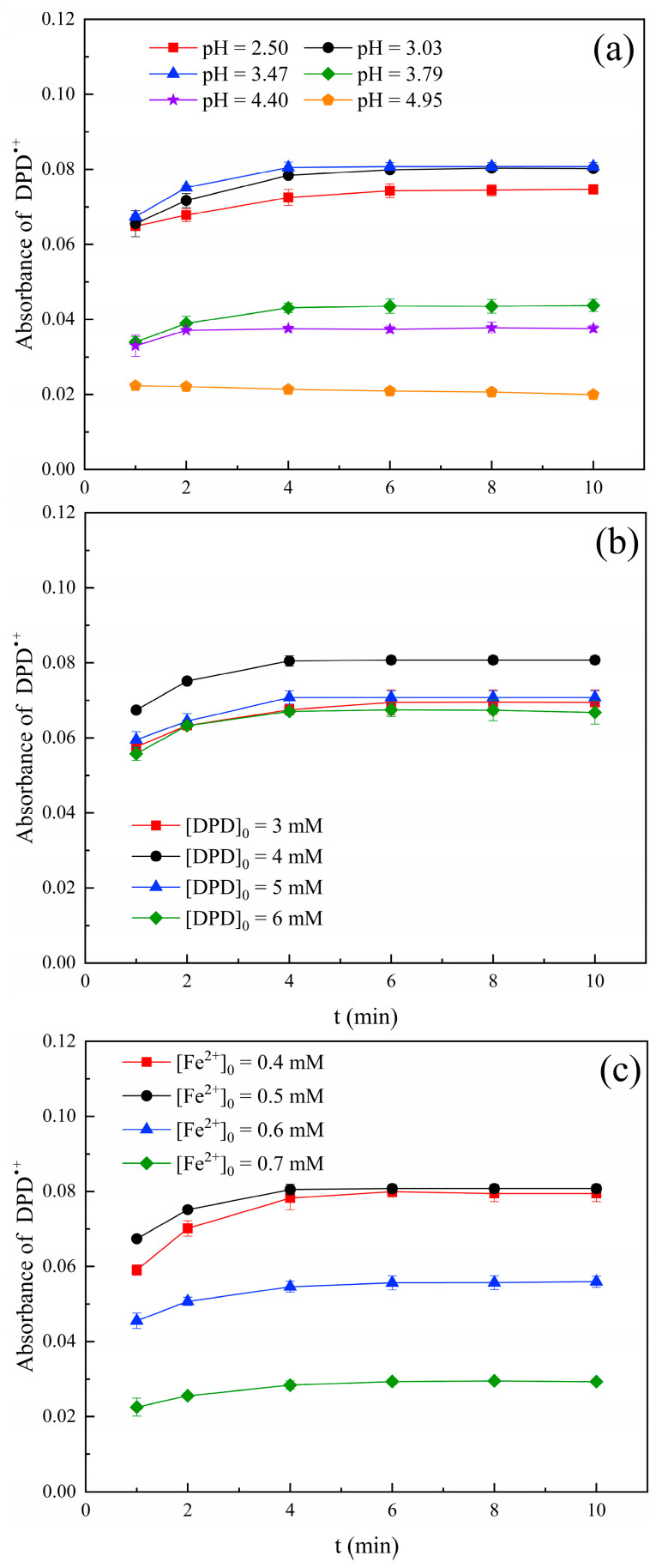
(**a**) Effect of solution pH, (**b**) DPD concentration, and (**c**) Fe^2+^ concentration on the formation of DPD^•+^ when using 40 μM SPC. ((**a**): [DPD]_0_ = 4 mM and [Fe^2+^]_0_ = 0.5 mM; (**b**): [Fe^2+^]_0_ = 0.5 mM and pH value = 3.50 (in 50 mM PBS); and (**c**): [DPD]_0_ = 4 mM and pH value = 3.50. T = 25 °C).

It has been reported that pH value had an important impact on the generation process and stability of the DPD^•+^ [16]. The precipitation of iron hydroxide is formed when pH ≥ 5.0 [18]. Therefore, the effect of solution pH value on DPD^•+^ absorbance was investigated in this paper and the results are shown in Figure 2a. The absorbance increased when the pH value increased from 2.50 to 3.47. This was because H^+^ can react with ∙OH under strongly acidic conditions (pH < 3), resulting in a decreasing amount of ∙OH available for the oxidation of DPD; therefore, the production of DPD^•+^ was reduced [19]. The absorbance value decreased when the pH value increased from 3.47 to 4.95. This was determined by the existing forms of iron species. Fe^2+^ could easily be converted into Fe(III), and the reaction rate of Fe(III) with H_2_O_2_ (0.01 M^−1^ cm^−1^, Equation (4)) was far lower than that of Fe^2+^ with H_2_O_2_ (76 M^−1^ cm^−1^) with the increase in the pH value [20]. Therefore, we can infer that the amount of Fe^2+^ used to activate SPC became less, which reduced the production of ∙OH and ultimately led to the formation of less DPD^•+^. Therefore, the optimal pH value was 3.50, and the optimal reaction time was 4 min since the absorbance of DPD^•+^ tended to be stable as of 4 min.

(4) $${\mathrm{Fe}}^{3+}{+H}_{2}O_{2}\;\to {\;\mathrm{Fe}}^{2+}{+\mathrm{HO}}_{2}∙{+H}^{+}$$

#### 2.2.2. Concentration of DPD

Figure 2b shows the effect of DPD concentration on the formation of DPD^•+^. The absorbance values were similar at DPD concentrations of 3 mM, 5 mM, and 6 mM, all of which were lower than that of 4 mM. Therefore, DPD = 4 mM was chosen as the appropriate concentration.

#### 2.2.3. Dosage of Fe^2+^

The dosage of Fe^2+^ affected the production of ∙OH; it was a key variable in the oxidation of DPD by the Fe^2+^/SPC system. The effect of Fe^2+^ concentration on the formation of DPD^•+^ is shown in Figure 2c. The absorbance value of DPD^•+^ increased with the increasing dosage of Fe^2+^ from 0.4 mM to 0.5 mM. This was because the lower concentration of Fe^2+^ failed to fully activate SPC and the yield of ∙OH became less. Therefore, the formation of DPD^•+^ was affected. The absorbance value of DPD^•+^ decreased significantly with the further increase in the dosage of Fe^2+^ to 0.7 mM. This was because excessive Fe^2+^ consumes H_2_O_2_ and inhibits the formation of DPD^•+^ (Equations (5)–(9)) [21]. In addition, the absorbance value of DPD^•+^ was stable as of 6 min for Fe^2+^ = 0.4 mM, while the absorbance value of DPD^•+^ was stable as of 4 min for Fe^2+^ = 0.5 mM. Therefore, 0.5 mM of Fe^2+^ was chosen as the optimal dosage.

(5) $${\mathrm{Fe}}^{3+}{+H}_{2}O_{2}\;\to \;\mathrm{Fe}{\left({\mathrm{HO}}_{2}\right)}^{2+}{+H}^{+}$$

(6) $$\mathrm{Fe}{\left({\mathrm{HO}}_{2}\right)}^{2+}{+H}_{2}O_{2}\;\to \;\mathrm{Fe}(\mathrm{OH}){\left({\mathrm{HO}}_{2}\right)}^{+}{+H}^{+}$$

(7) $$\mathrm{Fe}{\left({\mathrm{HO}}_{2}\right)}^{2+}\;\to {\;\mathrm{Fe}}^{2+}+{{\mathrm{HO}}_{2}}^{•}$$

(8) $${\mathrm{Fe}}^{2+}{{+\mathrm{HO}}_{2}}^{•}\;\to {\;\mathrm{Fe}}^{3+}{+\mathrm{HO}}_{2}^{-}\;$$

(9) $${\mathrm{Fe}}^{2+}+∙\mathrm{OH}\;\to {\;\mathrm{Fe}}^{3+}{+\mathrm{OH}}^{-}$$

In summary, the optimal experimental conditions for the Fe^2+^/SPC-DPD spectrophotometry were as follows: pH value 3.50, 4 mM of DPD, 0.5 mM of Fe^2+^, and 4 min of reaction time. The subsequent experiments were all carried out under these conditions unless otherwise stated. In addition, the effects of the above three variables (pH value and concentration of DPD and Fe^2+^) on the formation and stabilization of DPD^•+^ were also investigated with the SPC concentration of 20 μM (Figure S1). According to the results, the absorbance value of DPD^•+^ at the SPC concentration of 40 μM was about twice that of 20 μM when other experimental conditions were the same, and the same optimal experimental conditions could be concluded, which further confirmed the experimental results.

### 2.3. Method Evaluation

#### 2.3.1. Calibration Curve of SPC

**Figure 3:**
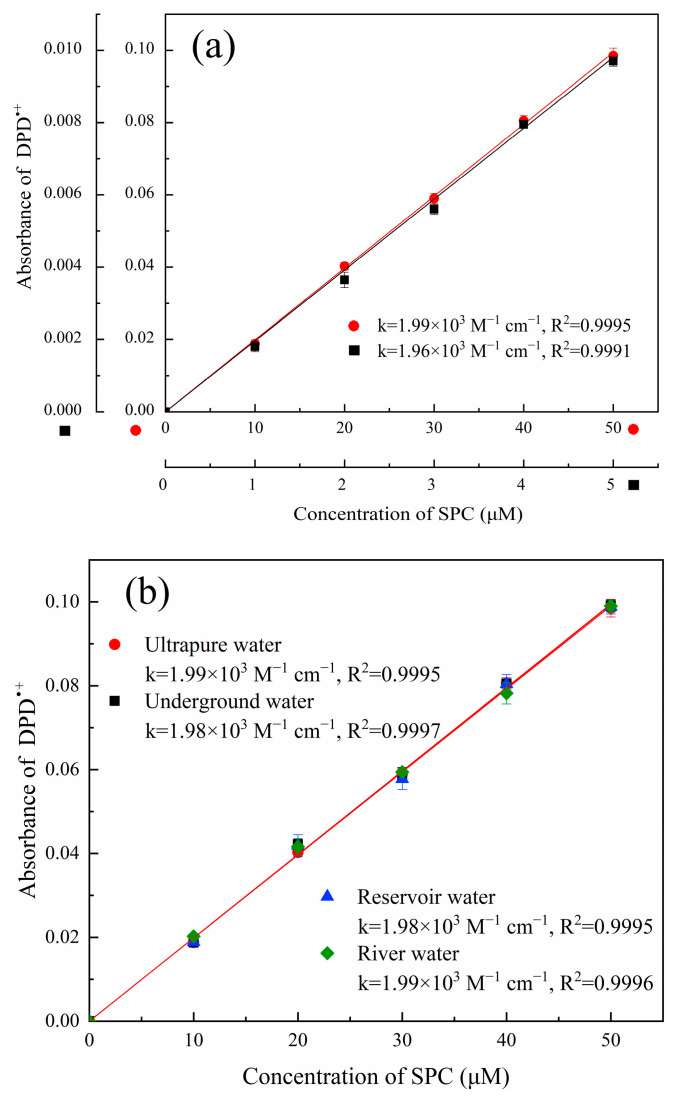
(**a**) SPC concentration calibration curves in ultrapure water in the SPC concentration ranges of 0–50 μM (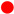) and 0–5 μM (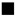); (**b**) calibration curves for the determination of SPC in natural waters. ([DPD]_0_ = 4 mM, [Fe^2+^]_0_ = 0.5 mM, pH value = 3.50, t = 4 min, and T = 25 °C).

The calibration curves for the determination of SPC via Fe^2+^/SPC-DPD spectrophotometry are shown in Figure 3a. The calibration curves conducted in two different SPC concentration ranges (0–5 μM and 0–50 μM) showed good linearity (R^2^ > 0.9990), and the slopes of the calibration curves were almost the same. Moreover, the SPC solution was prepared with natural underground water, reservoir water, and river water instead of ultrapure water. The SPC calibration curve was drawn to evaluate the effect of the natural water matrix (Figure 3b). The results showed that the three calibration curves displayed good linearity (R^2^ > 0.9990) and they had almost similar slopes to ultrapure water.

#### 2.3.2. DL and QL

The DL and QL of this method in ultrapure water were 0.7 and 2.5 μM, respectively (Table S2). Meanwhile, the DL and QL of this method in natural waters were in the range of 0.9–1.0 and 3.0–3.3 μM, respectively, which were slightly higher than that of ultrapure water. This indicated that the proposed Fe^2+^/SPC-DPD spectrophotometry method had low interference, and was hardly affected by the natural water matrix.

#### 2.3.3. Recovery Rate

A certain amount of SPC was added to the sample and the obtained results were compared with the theoretical values to evaluate the accuracy of the Fe^2+^/SPC-DPD spectrophotometry. Different concentrations of SPC (2–35 μM) were added to ultrapure water and three were added to natural water, and then the blank spiked recoveries of SPC in different waters were measured. It can be seen from Table S3 that the recovery rates of SPC were acceptable (95–105%). Therefore, it could be concluded that the Fe^2+^/SPC-DPD spectrophotometry had good accuracy for the determination of trace concentration of SPC in various waters.

#### 2.3.4. Assessment of the Stability of DPD^•+^

The evaluation of DPD^•+^ stability was conducted by examining the variation in absorbance values in different waters. Overall, the absorbance values of DPD^•+^ varied by 3.95% on average in different waters (Figure S2), indicating that DPD^•+^ was relatively stable. Liu et al. [16] found the DPD^•+^ generated in ultrapure water changed by 2.5% within 30 min when DPD was used to indicate permanganate concentration. This indicated that the DPD^•+^ had strong stability and was an ideal indicator.

### 2.4. Effect of Hydroxylamine

**Figure 4:**
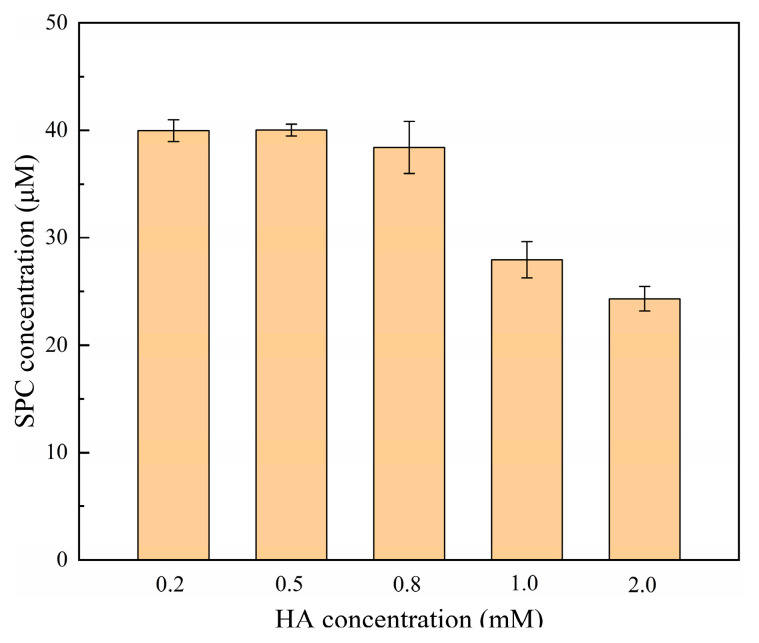
Effect of HA on the determination of SPC using the Fe^2+^/SPC-DPD spectrophotometry method. ([DPD]_0_ = 4 mM, [Fe^2+^]_0_ = 0.5 mM, [SPC]_0_ = 40 μM, pH value = 3.50, t = 4 min, and T = 25 °C).

Hydroxylamine (HA) is a strong reducing agent. In recent years, researchers have focused on the application of HA to improve the Fenton or Fenton-like degradation efficiency of pollutants [22,23] since the Fe^3+^/Fe^2+^ redox cycle can be enhanced by HA to ensure a stable supply of Fe^2+^ (Equation (10)) [24]. However, HA was previously reported to interfere with the determination of persulfate concentration via iodometric spectrophotometry [25]. This was because HA could reduce I_2_ to I^−^, which led to a decrease in the amount of I^3−^. It can be speculated that the addition of HA may cause the reduction reaction of DPD^•+^, thereby interfering with the determination of SPC concentration. Therefore, it is necessary to examine the effect of HA on SPC determination using the method proposed in this work (Figure 4).

As can be seen from Figure 4, HA at a concentration less than 0.8 mM had little effect on the determination of SPC, and the HA in the concentration range of 1–2 mM had great interference with the determination of SPC. This was because the high concentration of HA could compete with DPD for ∙OH generated from the decomposition of SPC, thus reducing the formation of DPD^•+^ (Equation (11)) [26]. As a result, this approach worked well for the analysis of SPC with HA less than 0.8 mM.

(10) $${\mathrm{NH}}_{3}{\mathrm{OH}}^{+}{+\mathrm{Fe}}^{3+}\;\to {\;\mathrm{Fe}}^{2+}+\mathrm{nitrogenous}\;\mathrm{products}\;$$

(11) $${\mathrm{NH}}_{3}{\mathrm{OH}}^{+}+∙\mathrm{OH}\;\to {\;\mathrm{OH}}^{-}+\mathrm{nitrogenous}\;\mathrm{products}\;$$

### 2.5. Utilization Rate of SPC in SPC-Based AOPs

**Figure 5:**
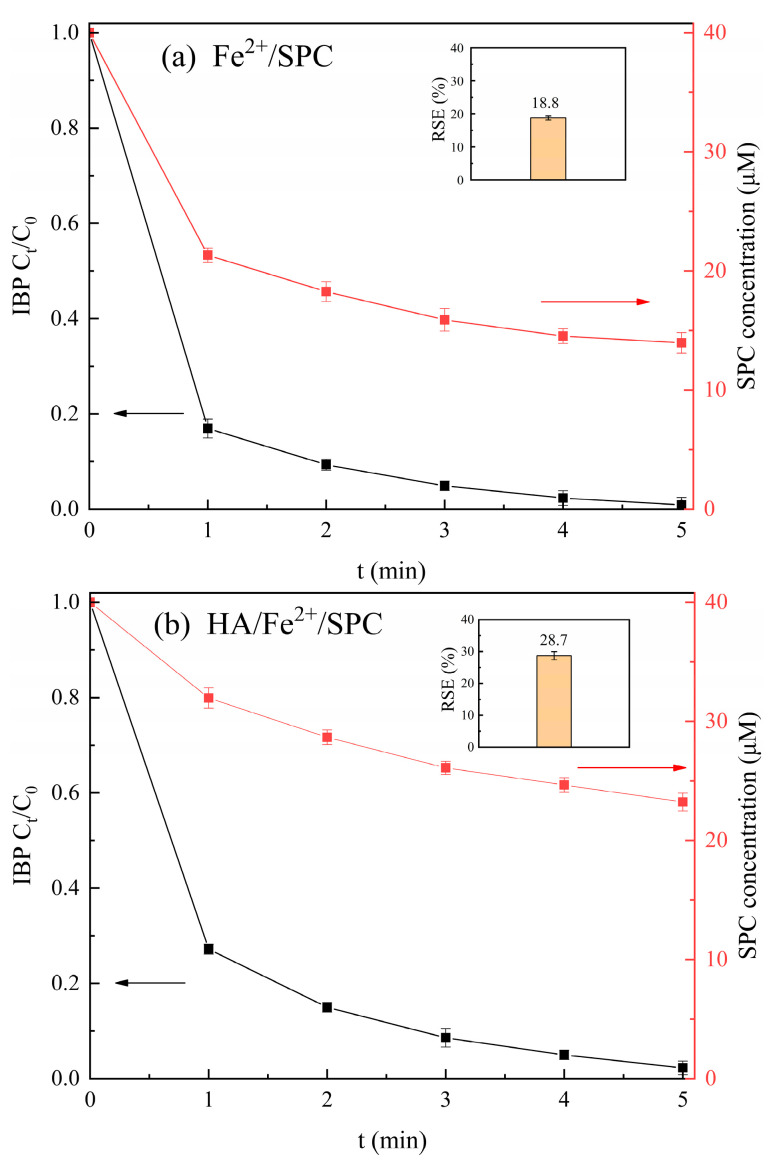
The degradation of ibuprofen and decomposition of SPC in the Fe^2+^/SPC system (**a**) and the HA/Fe^2+^/SPC system (**b**). (**a**) [Fe^2+^]_0_ = 0.5 mM, [SPC]_0_ = 40 μM, and [ibuprofen]_0_= 5 μM. (**b**) [Fe^2+^]_0_ = 50 μM, [SPC]_0_ = 40 μM, [HA]_0_ = 0.1 mM, and [ibuprofen]_0_= 5 μM). pH value = 3.00 in the IBP degradation systems and pH value = 3.50 in the SPC concentration measurement. The inset indicates the calculated RSE values (%).

The Fe^2+^/SPC-DPD spectrophotometry proposed in this work was used to determine the SPC concentration to assess the reaction stoichiometric efficiency (RSE) of the SPC-based AOPs, which is important to explore the activation methods of SPC. In this work, the variation in SPC concentration was observed during the degradation of ibuprofen (a representative pharmaceutical contaminant) by two SPC-based AOPs, and the RSE was calculated (Equation (12)) [27]. The results are shown in Figure 5.

(12) $$\mathrm{RSE}\;\left(\%\right)=\mathrm{the}\;\mathrm{number}\;\mathrm{of}\;\mathrm{moles}\;\mathrm{of}\;\frac{\mathrm{Ibuprofen}\;\mathrm{degraded}}{\mathrm{SPC}\;\mathrm{consumed}}\;\times \;100\%\;$$

As shown in Figure 5a, the concentration of SPC decreased rapidly from 40 μM to 21.3 μM at 1 min in the Fe^2+^/SPC system, and then it continued to decrease within 5 min, with 65% of SPC utilized at 5 min. Meanwhile, ibuprofen was rapidly degraded by 83% at 1 min in the Fe^2+^/SPC system, and then degraded by 100% at 5 min. This was because Fe^2+^ was enough at the beginning of the reaction, and the ·OH generated from Fe^2+^-activated SPC can rapidly oxidize ibuprofen. As the reaction progressed, Fe^2+^ was converted into Fe^3+^, but the interaction between Fe^3+^ and H_2_O_2_ was relatively slow [28]. Therefore, the effective concentration of Fe^2+^ decreased and the synthesis of ·OH slowed down, resulting in a gradual slowdown in the degradation of ibuprofen.

As shown in Figure 5b, the required Fe^2+^ dosage in the HA/Fe^2+^/SPC system was only one-tenth of the Fe^2+^/SPC system. However, ibuprofen was also completely degraded in 5 min, and the utilization rate of SPC was high (42%). Figure 5b also showed that SPC was slowly consumed in the HA/Fe^2+^/SPC system. This was primarily caused by the addition of HA, which quickly reduced Fe^3+^ to Fe^2+^, essentially ensuring the availability of Fe^2+^ [29].

Furthermore, it can be seen from Figure 5 that the RSE value for the reaction of ibuprofen with SPC in the Fe^2+^/SPC and HA/Fe^2+^/SPC systems were 19% and 29%, respectively, which meant that 1 mol of ibuprofen consumed 5.32 mol and 3.48 mol of SPC in the Fe^2+^/SPC and HA/Fe^2+^/SPC systems, respectively. The RSE was slightly higher in HA-containing systems; therefore, from the perspective of the efficient utilization of SPC, Fe^2+^/SPC was inferior to the HA/Fe^2+^/SPC system. Zou et al. [26] and Dai et al. [30] established the iodine spectrophotometry method to determine the concentrations of PDS and H_2_O_2_ in water and compared the RSE of Fe^2+^/PDS (3.9%), HA/Fe^2+^/PDS (12.2%), Fe^2+^/H_2_O_2_ (5.2%), and HA/Fe^2+^/H_2_O_2_ (5.4%) systems to degrade diclofenac. They concluded that HA-containing systems had a high utilization of oxidants, which was consistent with our conclusion.

### 2.6. Toxicity Assessment

**Figure 6:**
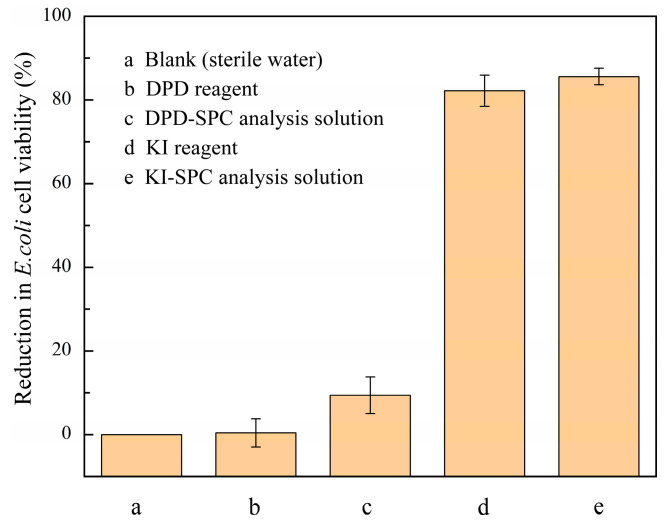
Reduction in *E. coli* cell viability via Fe^2+^/SPC-DPD spectrophotometry and iodometric spectrophotometry methods. (**a**) sterile water; ((**b**) DPD reagent. [DPD]_0_ = 4 mM; (**c**) DPD-SPC analysis solution. [DPD]_0_ = 4 mM, [SPC]_0_ = 40 μM, [Fe^2+^]_0_ = 0.5 mM, pH value = 3.50, and t = 4 min; (**d**) KI reagent. [KI]_0_ = 0.8 mM; and (**e**) KI-SPC analysis solution. [KI]_0_ = 0.8 mM and [SPC]_0_ = 40 μM. T = 25 °C).

It has been reported that the wastewater generated from the determination of oxidant may affect the cell activity of *E. coli*, causing a negative environmental impact [31]. Therefore, the toxicity of the wastewater from the Fe^2+^/SPC-DPD spectrophotometry proposed in this work and the iodometric spectrophotometry reported in previous literature were both evaluated [10]. The toxicity of *E. coli* to solutions related to these two methods is shown in Figure 6. The calibration curve for the determination of SPC via iodometric spectrophotometry is shown in Figure S3, and the specific experimental method is described in the Supplementary Materials.

It can be seen from Figure 6. that the DPD solution had almost no inhibitory effect on the cellular activity of *E. coli* (0.4%). The wastewater produced during the determination of SPC via Fe^2+^/SPC-DPD spectrophotometry had no significant impact on the cellular activity of *E. coli* (9.4%) either. As reported in previous literature [32], when the percentage reduction in *E. coli* cell viability was less than 10%, the wastewater was considered non-toxic and could be safely deposed. However, although the concentration of KI solution (0.8 mM) was five times lower than the DPD concentration (4 mM), both the KI solution and the wastewater generated by the iodometric spectrophotometry showed significant inhibition of *E. coli* (82% and 86%, respectively). Therefore, it can be concluded that Fe^2+^/SPC-DPD spectrophotometry is a safe and environmentally friendly method.

## 3. Materials and Methods

### 3.1. Experimental Material

The experimental materials used in this work are listed in the Supplementary Materials (SM).

### 3.2. Experimental Methods

#### 3.2.1. SPC Calibration Curve

An amount of 10 mL of phosphate buffer solution (PBS) (125 mM, pH 3.50), 5 mL of DPD solution (20 mM), 5 mL of ferrous sulfate solution (2.5 mM), and 5 mL of SPC solution (0–250 μM) were mixed together and reacted for 4 min, and then the mixed solution was transferred into a 1 cm cuvette. The mixed solution without SPC was used as a reference. The absorbance values of the samples at wavelengths of 510 nm and 551 nm were measured via UV-Vis spectrophotometry (SPECORD 200, Analytik Jena AG, Jena, Germany), and all experiments were repeated three times. The calibration curve was found to be between the SPC concentration and the absorbance value of DPD^•+^. SPC concentration can be calculated according to Equation (13) by taking the detected wavelength of 551 nm, for example:

(13) $${\left[\mathrm{SPC}\right]}_{\mathrm{sample}}=\frac{A_{551\mathrm{nm}\;}^{l}V_{\mathrm{final}}}{{\varepsilon \;\gamma \;l\;V}_{\mathrm{sample}}}$$

$A_{551\mathrm{nm}}^{l}$: the absorbance value of DPD^•+^ at 551 nm;

V_final_: the volume of mixed solution (25 mL);

ε: molar absorption coefficient for DPD^•+^ (21,000 M^−1^ cm^−1^ [13]);

l: the size of the cuvette (1 cm);

V_sample_: the volume of the sample (5 mL);

γ: the reaction stoichiometric coefficient between SPC and DPD (γ = k/ε) [12].

#### 3.2.2. Detection Limit and Quantitative Limit

The detection limit (DL) and the quantitative limit (QL) of this method were calculated according to Equations (14) and (15) [33]:

(14) $$\mathrm{DL}=\frac{3\sigma }{k}$$

(15) $$\mathrm{QL}=\frac{10\sigma }{k}$$

where σ is the standard deviation of the six blank samples; k is the slope of the calibration curves.

#### 3.2.3. Recovery Rate

The same solution of PBS, DPD, and ferrous sulfate used in Section 2.2.1 and 5 mL of SPC solution (10–350 μM) were mixed together and reacted for 4 min and then analyzed following the same procedure as in Section 2.2.1. All experiments were repeated six times. The blank spiked recovery rate of the sample was calculated using the ratio of the measured value and the theoretical value.

#### 3.2.4. DPD^•+^ Stability Evaluation

The same solution of PBS, DPD, and ferrous sulfate used in Section 2.2.1 and 5 mL of SPC solution (200 μM) were mixed together and reacted for 4 min and then analyzed following the same procedure as in Section 2.2.1. The stability of the generated DPD^•+^ was explored, the absorbance was measured every 0.5 min within 4–20 min, and the absorbance variation in DPD^•+^ was expressed by the ratio of absorbance at *t* min with that at 4 min.

### 3.3. Degradation of Ibuprofen by SPC-Based AOPs

Fe^2+^/SPC and HA/Fe^2+^/SPC systems were selected as representatives of SPC-based AOPs, the concentration of SPC during the degradation of ibuprofen by the two AOPs was determined, and the utilization rate of SPC was calculated. The experimental details and the determination method of ibuprofen can be found in the SM.

### 3.4. E. coli Cell Activity Analysis

*E. coli* was inoculated in LB liquid medium and incubated in a constant temperature incubator (ZQPL-200, Tianjin Leibo Terry Equipment Co., Ltd., Tianjin, China) at 37 °C for 12 h, and then centrifuged three times and washed using sterile saline (0.8%); the centrifuge (TGL20M, KaiT Co. Ltd., Shanghai, China) was set at 8000 rpm for 5 min. Finally, the cleaned *E. coli* was re-suspended in sterile saline and the absorbance at 600 nm (OD_600_) was determined via UV-Vis spectrophotometry to ensure that the signal was in the range of 0.10–0.65 and stored in the refrigerator.

The following samples (25 mL) were prepared: (1) sterile water; (2) DPD reagent; (3) DPD-SPC analysis solution; (4) KI reagent; and (5) KI-SPC analysis solution. An amount of 0.25 mL of *E. coli* solution was added to the above five samples and incubated in a constant temperature shaker at 25 °C, 180 r/min, for 3 h and then the OD_600_ was measured. The inhibition rate of *E. coli* cell activity can be calculated based on Equation (16):

(16) $$I=\frac{A_{0}{-A}_{t}}{A_{0}}\;\times \;100\%$$

where A_0_ and A_t_ represent the absorbance of *E. coli* suspensions in the blank and experimental groups, and the experiments were conducted three times.

## 4. Conclusions

A novel UV-Vis spectrophotometric method was successfully developed to determine SPC concentration in water since SPC can oxidize DPD to DPD^•+^ with the presence of Fe^2+^, and the absorbance of DPD^•+^ at 551 nm can be used to calculate SPC concentration. The proposed method had many advantages, such as good linearity and recovery rate, acceptable detection and quantitative limit, easy operation, less interference, and environmental friendliness, and it is promising in the practical determination of SPC in aqueous solution.

## Data Availability

Data are available upon reasonable request.

## Supplementary Materials

The following supporting information can be downloaded at: https://www.mdpi.com/article/10.3390/molecules28237732/s1. Supplementary file contains Tables S1–S3 and Figures S1–S3. Table S1: Water quality parameters of natural water samples. Table S2: DL and QL of the Fe(II)/SPC-DPD spectrophotometric method for determination of SPC in different water matrices (n = 6). Table S3: Spiked recoveries of SPC measured in different types of water (n = 6) ([DPD]0 = 4 mM, [Fe2+]0 = 0.5 mM, pH value = 3.50, t = 4 min, and T = 25 °C). Figure S1: (a) Effect of solution pH, (b) DPD concentration, and (c) Fe^2+^ concentration on the formation of DPD^•+^ when using 20 μM SPC ((a): [DPD]0 = 4 mM and [Fe2+]0 = 0.5 mM; (b): [Fe2+]0 = 0.5 mM and pH value = 3.50; (c): [DPD]0 = 4 mM and pH value = 3.50. T = 25 °C). Figure S2: Stability of DPD•+ in different waters ([DPD]0 = 4 mM, [Fe2+]0 = 0.5 mM, [SPC]0 = 40 μM, pH value = 3.50, t = 4 min, and T = 25 °C). Figure S3: Calibration curve for the determination of SPC concentration by iodometric method at 352 nm.
