# Supplementary material for: A Novel Spectrophotometric Method for Determination of Percarbonate by Using N, N-Diethyl-P-Phenylenediamine as an Indicator and Its Application in Activated Percarbonate Degradation of Ibuprofen
Source: Molecules. 2023 Nov 23;28(23):7732. doi: 10.3390/molecules28237732 (PMC10708432; doi:10.3390/molecules28237732)
Supplement: Supplementary file 1 [file molecules-28-07732-s001.zip › molecules-2727530-supplementary.pdf]

Supporting information

A novel spectrophotometric method for determination of percarbonate by using N, N-diethyl-p-phenylenediamine as an indicator

Jinying Li, Aoxue Chen, Qingling Meng, Honghai Xue*, Baoling Yuan

Key Laboratory of Songliao Aquatic Environment, Ministry of Education, Jilin Jianzhu University, Changchun, 130118, P. R. China.

Corresponding author

Honghai Xue, E-mail: xuehonghai@jlju.edu.cn

Contact information of other authors

Jinying Li, E-mail: 1986472643@qq.com

Aoxue Chen, E-mail: chenaoxue2002@163.com

Qingling Meng, E-mail: mengql1984@163.com

Baoling Yuan, E-mail: yuanbl@hotmail.com

Total pages: 7, including cover page

Number of Figures: 3

Number of Tables: 3

1. Chemicals

Sodium percarbonate (CAS 15630-89-4, molecular weight 157.01, purity 98%), N, N-diethyl-p-phenylenediamine sulfate salt (CAS 6065-27-6, molecular weight 262.33, purity 98%), hydroxylamine sulfate (CAS 10039-54-0, molecular weight 164.14, purity 99%), and ibuprofen (CAS 15687-27-1, molecular weight 206.28, purity 99%) were purchased from Aladdin Biochemical Technology Co. Ltd., Shanghai, China. Sodium hydroxide (CAS 1310-73-2, molecular weight 40, purity 96%), sodium bicarbonate (CAS 144-55-8, molecular weight 84.01, purity 99.5%), sodium dihydrogen phosphate dihydrate (CAS 13472-35-0, molecular weight 156.01, purity 99%), di-sodium hydrogen phosphate dodecahydrate (CAS 10039-32-4, molecular weight 358.14, purity 99%), ferrous sulfate heptahydrate (CAS 7782-63-0, molecular weight 278.01, purity 99%), potassium iodide (CAS 7681-11-0, molecular wight 166, purity 99%), phosphoric acid (CAS 7664-38-2, molecular weight 98, purity 85%) were supplied by Sinopharm Chemical Reagent Co. Ltd., China. Methanol (CAS 67-56-1, molecular weight 32.04, purity 99.9%) was obtained from Fisher Chemical, USA. LB liquid medium was purchased from the Nantong Kinghunt Biology Technological Development Co., Ltd., China. *Escherichia coli* (ATCC25922) was purchased from Shanghai Luwei Technology Co., Ltd., China. The water used in the experiments was ultrapure water (Hangzhou Wahaha Group Co. Ltd., China) unless otherwise stated.

Reservoir water was obtained from the Xinlicheng Reservoir in Changchun, China. Underground water was obtained from a village near the Jingyuetan National Scenic Area in Changchun, China. River water was obtained from the Xiaoheyanzi River in Changchun, China. Natural water samples were collected on November 28, 2022. They were stored at 4°C after filtration through a 0.45 μm membrane and used within 3 d. The water quality parameters of the natural water samples were given in Table S1.

Table S1 Water quality parameters of natural water samples

Water samples	pH value	Redox potential (mV)	DO (mg/L)	TN (mg/L)	TOC (mg/L)	COD(mg/L)
Underground water	6.34	558	9.25	5.25	5.08	54.18
Reservoir water	6.67	283	10.52	1.74	8.20	94.82
River water	6.63	495	9.10	3.04	7.30	82.78

Note: DO, TN, TOD, and COD refer to dissolved oxygen, total nitrogen, total oxygen carbon, and chemical oxygen demand, respectively.

2. Degradation of ibuprofen by Fe^{2+} /SPC and HA/Fe^{2+}/SPC

The degradation of ibuprofen by Fe^{2+}/SPC system was conducted as follows: 10 mL of ferrous sulfate solution (25 mM), 50 mL of ibuprofen solution (50 μM) and 436 ml of ultrapure water were added to a 500 mL beaker, and then 4 mL of SPC solution (5 mM) was added to the beaker and the solutions were mixed together under magnetic stirring. Phosphoric acid was used to adjust the pH of the mixed solution to 3.0. During the degradation process, 6 mL of sample was taken out every 1 min within 5 min. 5 mL sample was used for the determination of SPC concentration, and 1 mL of sample was used for the determination of IBP concentration.

The degradation of ibuprofen by HA/Fe^{2+}/SPC system was conducted as follows: 1 mL of ferrous sulfate solution (25 mM), 2 mL of hydroxylamine sulfate solution (25 mM), and 50 mL of ibuprofen solution (50 μM) and 443 ml of ultrapure water were added to a 500 mL beaker, and then 4 mL of SPC solution (5 mM) was added to the beaker. And the rest protocols were the same as that of the Fe^{2+}/SPC system.

For IBP analysis, 50 μL of methanol was added to the sample to stop the reaction, and the sample was filtered via a 0.22 μm membrane. The concentration of ibuprofen was determined by high-performance liquid chromatography equipped with a UV detector (1200 series, Agilent, USA). The detection parameters were as follows: A C18 symmetry column (5 μm, 4.6 × 150 mm) was utilized as the stationary phase with a column temperature of 40°C. 0.1% phosphoric acid and methanol were used as the eluents with a volume ratio of 25:75. The flow rate was 0.8 mL min^{-1}. The detection wavelength was 223 nm, and the injection volume was 100 μL.

3. Effect of pH value, DPD and Fe^{2+} concentrations on the generation of $DPD^{\bullet+}$ by using 20 μM SPC

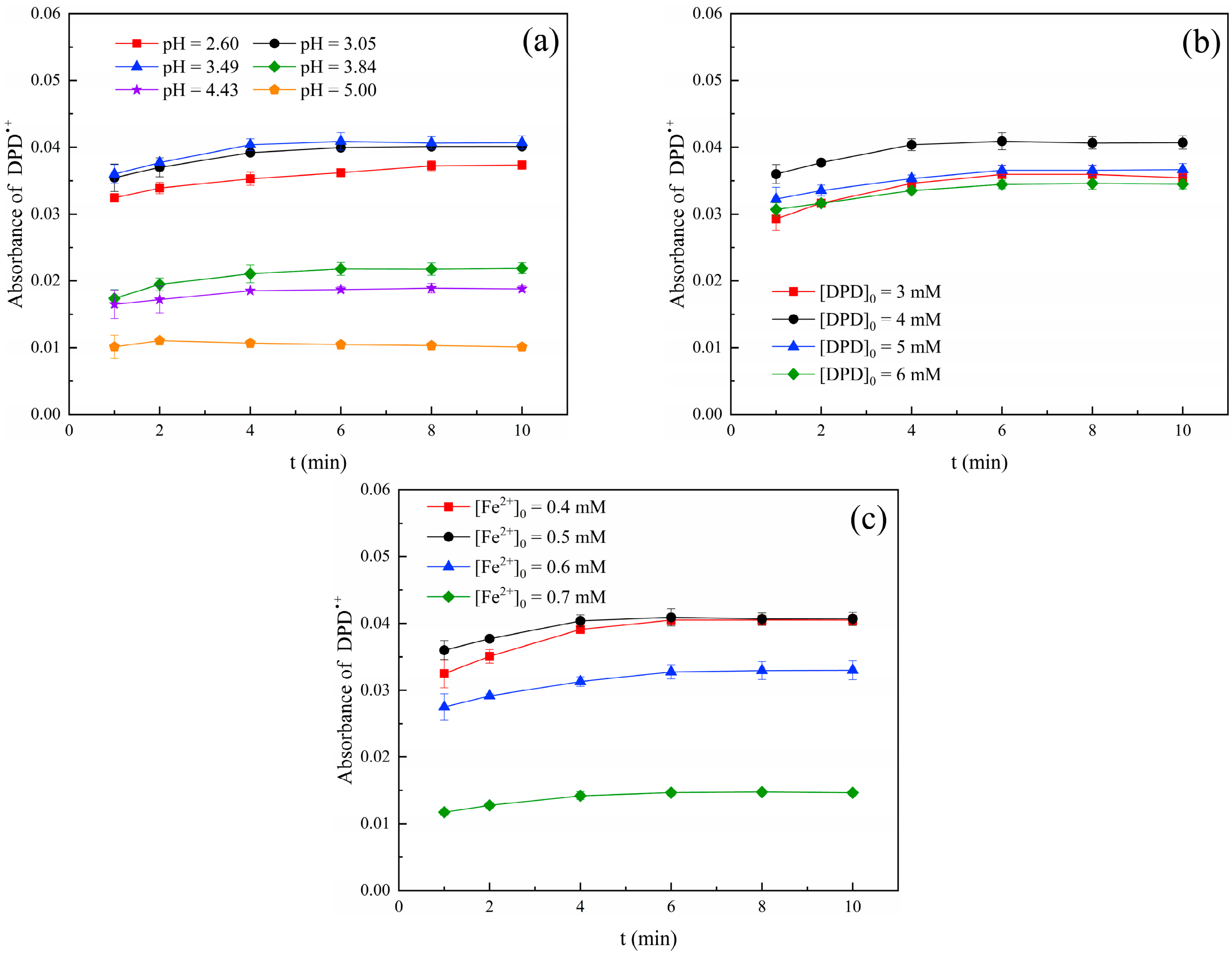

Figure S1. (**a**) Effect of solution pH, (**b**) DPD concentration, and (**c**) Fe^{2+} concentration on the formation of $DPD^{\bullet+}$ when using 20 μM SPC. ((**a**): $[DPD]_0$ = 4 mM and $[Fe^{2+}]_0$ = 0.5 mM; (**b**): $[Fe^{2+}]_0$ = 0.5 mM and pH value = 3.50; (**c**): $[DPD]_0$ = 4 mM and pH value = 3.50. T = 25 ℃)

4. Detection limit (DL) and quantitative limit (DL)

Table S2 DL and QL of the Fe(II)/SPC-DPD spectrophotometric method for determination of SPC in different water matrices (n=6)

Water samples	The absorbance of the blank samples at 551 nm						σ	k	DL (μM)	QL (μM)
Ultrapure water	0.0026	0.0022	0.0014	0.0015	0.0012	0.0018	0.000484	0.00198	0.73	2.45
Underground water	0.0024	0.0033	0.0020	0.0025	0.0017	0.0034	0.000624	0.00197	0.95	3.17
Reservoir water	0.0025	0.0016	0.0022	0.0010	0.0030	0.0016	0.000659	0.00198	0.99	3.33
River water	0.0034	0.0019	0.0021	0.0017	0.0029	0.0020	0.000607	0.00201	0.91	3.02

5. Recovery rate

Table S3 Spiked recoveries of SPC measured in different types of water (n = 6). ($[DPD]_0$ = 4 mM, $[Fe^{2+}]_0$ = 0.5 mM, pH value = 3.50, t = 4 min, and T = 25 ℃)

Water samples	SPC concentration (μM)		Recovery rate (%)	Water samples	SPC concentration (μM)		Recovery rate (%)
	Spiked	Measured			Spiked	Measured	
Ultrapure water	2.00	1.91	95.35	Reservoir water	2.00	1.91	95.56
	8.00	7.61	95.16		8.00	8.14	101.81
	15.00	15.69	104.60		15.00	14.86	99.04
	25.00	25.23	100.93		25.00	25.37	101.49
	35.00	35.51	101.46		35.00	36.64	104.69
Underground water	2.00	1.96	98.04	River water	2.00	1.96	97.92
	8.00	8.21	102.65		8.00	7.98	99.80
	15.00	15.67	104.44		15.00	15.58	103.83
	25.00	24.84	99.34		25.00	25.13	100.50
	35.00	34.15	97.56		35.00	34.38	98.21

6. Assessment of the stability of $DPD^{\bullet+}$

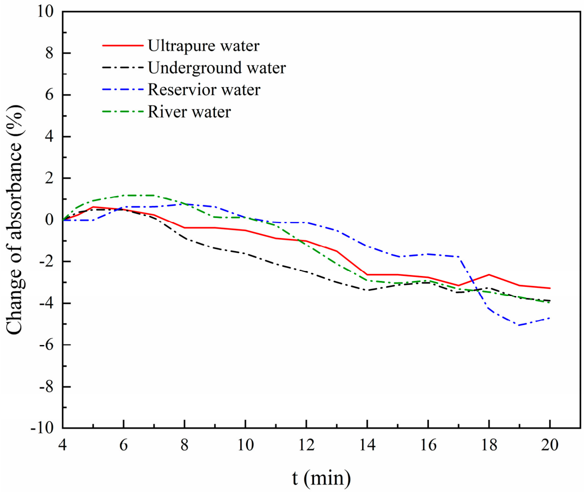

Figure S2. Stability of $DPD^{\bullet+}$ in different waters. ($[DPD]_0$ = 4 mM, $[Fe^{2+}]_0$ = 0.5 mM, $[SPC]_0$ = 40 μM, pH value = 3.50, t = 4 min, and T = 25 ℃)

7. Iodometric spectrophotometric determination of SPC concentration

The specific experimental procedures of iodometric spectrophotometry were as follows: A stock solution was prepared by dissolving 4.0 g KI and 0.2 g $NaHCO_3$ in 40 mL of ultrapure water. 100 μL of different concentrations of SPC (10, 20, 30, 40, and 50 μM) were mixed with 4 mL of the stock solution, reacted for 10 min, and then transferred into a 1 cm cuvette. The absorbance of the solution at 352 nm was determined immediately by using ultrapure water as a reference. Based on the absorbance values and the corresponding SPC concentrations, the standard curve of the SPC was plotted, as shown in Fig. S2.

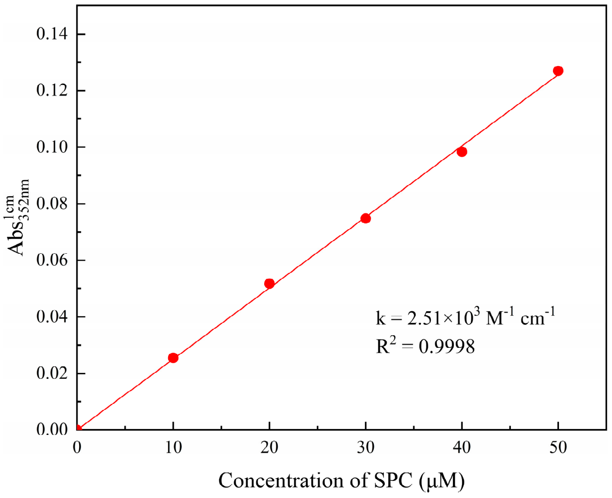

Figure S3. Calibration curve for the determination of SPC concentration by iodometric method at 352 nm
